# 

**Published:** 1826-09

**Authors:** 


### MONTHLY LIST OF MEDICAL BOOKS.

[*No books can be entered on this List except those sent to us for the purpose; as, in the lists hitherto transmitted, the names of works have frequently been given as published, which have not appeared for weeks, or even months, after.*]

A Treatise on a Modified Application of Moxa in the Treatment of Stiff and Contracted Joints, and also in Chronic Rheumatism, Rheumatic Gout, Lumbago, Sciatica, Indolent Tumors, &c. &c. Illustrated by Cases and Plates, &c. &c. Second Edition. By James Boyle, Esq. &c.—Pp. 219. 1826.

Principles of Dental Surgery, exhibiting a new Method of treating the Diseases of the Teeth and Gums, &c. In two Parts. By Leonard Koecker, Surgeon-Dentist, &c.—Pp. 445. London, 1826.

Part III. of a Series of Elementary Lectures on the Veterinary Art; wherein the Anatomy, Physiology, and Pathology of the Horse are essayed on the general Principles of Medical Science. By W. Percival, Esq. Member of the Royal College of Surgeons, &c.—Pp. 502. London, 1826.

Modern Domestic Medicine; or, a Popular Treatise illustrating the Character, Symptoms, Causes, Distinction, and correct Treatment, of all Diseases incident to the Human Frame, &c. To which is added, a Domestic Materia Medica, &c. &c. By Thomas John Graham, m.d. &c.—Pp. 561. London, 1826.

The London Medical and Physical Journal *professedly contains an account of the most important improvements which are made in Medicine and the collateral branches of Science: and it has been thought that, in addition to the means hitherto adopted for conveying this information, considerable advantage would be deduced from giving each month a history of such* Cases *occurring at* Public Institutions, *as might seem calculated to illustrate any points in pathology or practice. On mentioning this idea to the Physicians and Surgeons connected with some of the principal Hospitals and Dispensaries in the metropolis, the utmost readiness was manifested by these gentlemen to have an account of the cases under their care laid before the public; while every facility was promised for obtaining the necessary information. The Editor begs respectfully to express his sense of the liberality which has thus been shown by his professional brethren; and, while he absolves them from all responsibility with regard to the accuracy of the details, he pledges himself to take every means in his power of ascertaining the authenticity of the cases which he publishes; at the same time he requests it to be understood, that he will be ready to correct any misstatement, should such inadvertently be made.*

*The manner in which it appears to the Editor most eligible to arrange these, is according to subjects,—giving in succession a few cases of the same disease, particularly where there is any difference in the symptoms or treatment, the detail of which is likely to prove instructive. It is obvious that this object cannot be accomplished in every instance; but, with a view to secure its fulfilment as frequently as possible, it is intended to make the Reports retrospective, not confining them exclusively to those cases which are of recent occurrence.*

---

### NOTICE TO CORRESPONDENTS.

*Our Correspondents are respectfully requested, in drawing up Cases, to confine themselves as much as possible to matters which are essential, as we have been reluctantly compelled to decline several Communications on account of the minuteness of the details.*

*It is our intention hereafter, in acknowledging the receipt of Papers, to omit the authors' names in all those instances where the Communication is not suited for publication in this Journal.*

*Several Papers, acknowledged in the last two Numbers, will be published in the next.*

*The Communications of* Dr. Whitlock Nichol, Dr. Venables, Mr. Chevalier, Mr. Hamilton, Mr. Scot, Mr. Waller, Dr. Granville, Mr. Herapoth, Dr. Otto, *and* "A Licentiate:" *some of them will be found in the present Number.*

*We have answered the Communication from* Dr. Hoeker, *of Berlin, through the channel pointed out by him. The Numbers of his Journal to which he alludes have not been received.*

*We refer* Mr. H. *of Bristol, to page 293 of the present Number for the information he desires.*

Mr. Professor Mackenzie, *of Glasgow, is informed that his Paper has been unavoidably postponed. We shall give it in our next.*

